# Modeling the emergence of affective polarization in the social media society

**DOI:** 10.1371/journal.pone.0258259

**Published:** 2021-10-11

**Authors:** Petter Törnberg, Claes Andersson, Kristian Lindgren, Sven Banisch

**Affiliations:** 1 Amsterdam Institute for Social Science Research, University of Amsterdam, Amsterdam, The Netherlands; 2 Complex Systems Group, Physical Resource Theory, Chalmers University of Technology, Gothenburg, Sweden; 3 European Centre for Living Technology, University of Venice Ca’ Foscari, Venice, Italy; 4 Max Planck Institute for Mathematics in the Sciences, Max Planck Gesellschaft, Leipzig, Germany; St John’s University, UNITED KINGDOM

## Abstract

Rising political polarization in recent decades has hampered and gridlocked policymaking, as well as weakened trust in democratic institutions. These developments have been linked to the idea that new media technology fosters extreme views and political conflict by facilitating self-segregation into “echo chambers” where opinions are isolated and reinforced. This opinion-centered picture has recently been challenged by an emerging political science literature on “affective polarization”, which suggests that current polarization is better understood as driven by partisanship emerging as a strong social identity. Through this lens, politics has become a question of competing social groups rather than differences in policy position. Contrary to the opinion-centered view, this identity-centered perspective has not been subject to dynamical formal modeling, which generally permits hypotheses about micro-level explanations for macro-level phenomena to be systematically tested and explored. We here propose a formal model that links new information technology to affective polarization via social psychological mechanisms of social identity. Our results suggest that new information technology catalyzes affective polarization by lowering search and interaction costs, which shifts the balance between centrifugal and centripetal forces of social identity. We find that the macro-dynamics of social identity is characterized by two stable regimes on the societal level: one *fluid regime*, in which identities are weak and social connections heterogeneous, and one *solid regime* in which identities are strong and groups homogeneous. We also find evidence of hysteresis, meaning that a transition into a fragmented state is not readily reversed by again increasing those costs. This suggests that, due to systemic feedback effects, if polarization passes certain tipping points, we may experience run-away political polarization that is highly difficult to reverse.

## Introduction

Politics around the world has in recent decades entered an era of unprecedented political polarization, with harshening public discourse, growing divides between political camps, and diminishing shared political ground. This has had severe consequences for democracies around the world, bringing a weakening of trust of democratic institutions and norms, exacerbating intolerance and discrimination, hampering and gridlocking policy-making, undermining the credibility of government, and fueling public disaffection with political parties [1].

These developments have been linked to the emergence of social media and new information technology [2], in particular through notions such as “echo chambers” or “filter bubbles” [3–8]. According to a common version of these hypotheses, new media technologies allow us to self-segregate to avoid the discomfort of having our views challenged. Once separated, bubbles of aligned viewpoints are then seen as causing a reinforcement of opinion into more and more extreme positions, which then drives political conflict [7–9]. This version of the echo chamber theory thus reflects a traditional understanding of polarization as characterized by growing differences in issue positions: our opinions diverge and become more extreme, which leads to intensifying conflict.

However, recent political science work has put this traditional understanding of polarization into question. Contemporary polarization is here understood in terms such as *affective* [10] or *social* polarization [11], where growing differences in opinion are secondary to a process of partisanship strengthening as a social identity, which then channels and politicizes more and more social identities in plural societies [10, 12–15]. In this view polarization transforms politics into a battle-ground of a larger culture war, in which we engage with politics via the mechanisms of social identity rather than via rational debate [16, 17]. While social identity has always played a role in politics, this literature suggests we have entered a new regime where partisan identity comes to engulf and align other social identities [10, 12, 18]. Rather than contests over policies, elections turn into struggles between competing groups separated by a fundamental sense of difference [19].

This new understanding of polarization and its underlying mechanisms begs revisiting the suggested link between new media technology and the emergence of political polarization. We ask: *what is the causal link between new media technology and affective polarization*? To answer this question, we construct a formal model of identity, based on social psychological processes that underpin affective polarization, that we use for exploring the effects of new media technologies relaxing exogenous constraints on interaction. The social psychological mechanisms that are proposed to underpin affective polarization are well understood on the micro-level of social psychology, but since political polarization ultimately happens on a societal level, this leaves an important explanatory gap between these micro-level mechanisms and the macro-level phenomena of societal change they purport to explain [20]. In this paper we aim to explore the potential in such an approach using formal modeling to help us untangle the complex causality and feedback loops that inevitably results [21–23]. The model suggests that new information and communication technology may have brought about a shift in the macro-dynamics of political identity by enabling us to come together with people from across the world around shared attributes, and thus shifting the balance of social identity formation.

To formulate the model, we begin with a brief review of the work in the two lines of research outlined above, as well as the social psychological mechanisms that the identity-driven explanation is based upon. We then introduce the model and its results before discussing our findings.

### New social media and opinion-driven polarization

The notions of “echo chambers” and “filter bubbles” describe variations on a dominant idea—in particular within mainstream discourse—about how polarization is causally linked to new media technology. The echo chamber suggests that polarization is in part driven by new media technology that allows us to avoid the discomfort of exposing ourselves to opposing ideas or opinions by choosing what people and information we wish to interact with. The “filter bubble” denotes the situation where this occurs algorithmically as digital media personalizes our news and information environment [24].

The causal mechanism of polarization suggested by this literature is, in other words, that lack of exposure to alternative views make our opinions more extreme, which, in turn, intensifies tensions and intergroup conflict [7, 8, 25, 26]. This explanation thus operates on the level of individuals, focusing on polarization as something that happens to people or groups, as they become drawn into a certain social context.

The most common version of the echo chamber model thus relies on a traditional lens of democratic politics, which views politics as a process where voters arrive at policy preferences through debate and deliberation, proceeding then to support the party whose policies best match these preferences [27]. Simply put, in this understanding, democracy proceeds from rational debate, to policy preferences, to the selection of a party. Under these conditions, parties will tend to increase their share of votes by adapting their policies to voter preferences, which will create forces for political moderation [28]. Political polarization in turn appears as a self-accelerating process of diverging policy preferences [10, 29–36]. This departure point is not only how the echo chamber literature views polarization, but has been an important foundation of political science, as well as the formal modeling of opinion dynamics [37–41].

Researchers have attempted to empirically confirm the echo chamber hypothesis by examining whether users are indeed avoiding exposure to alternative views on new media platforms. The results of these studies have however been mixed: it does seem that users are clustering together with likeminded peers, but at the same time, they do not seem to be shielded from ideas from opposing camps, as users relatively frequently interact with ideas and people with whom they disagree [3–6, 42].

The hypothesis also suffers from a more fundamental issue, as a debate has emerged within political science about whether the type of polarization described by this model is in fact taking place. Studies using survey data has led political scientists to question that a divergence of opinion in the electorate is in fact taking place, as the political positions of most voters remain moderate [43–45]. This points to an issue for not only the echo chamber hypothesis, but for the broader understanding of political polarization of which it is part, as the observed intensification of political conflict does not appear to be driven by a divergence of issue positions.

### Affective polarization and the social identity model of politics

While it is debatable whether polarization in policy position is taking place in the electorate, there are clear signs of another form of polarization: voters are describing their feelings for the opposing party and its voters as growing increasingly cold, to the point that many voters now say they would be unhappy if a family member married outside of party lines [46]. While the fraction of voters who identify as Republicans or Democrats remains similar, partisans now view one another more negatively [47], and the number of strong partisans have risen [48]. Political scientists have come to refer to this as “affective”, “social” or “pernicious” polarization [49–51].

This observation has brought a profound shift in thinking, as the current wave of political polarization is not seen primarily as a process of diverging opinions, but rather as a move into a qualitatively different political dynamic altogether. The affective polarization literature sees polarization as driven by deeply rooted mechanisms of group affiliation in human psychology, as individuals instinctively think of themselves as representing collective categories [13, 52]. The literature on affective polarization draws on a large body of work in social psychology that has shown humans to be quick to develop distinctions between in-group and out-group on the basis of the most trivial and arbitrary of shared characteristics, triggering aversion toward the out-group, and positive feelings for the in-group [52, 53]. The more salient the group is to the sense of personal identity, the stronger these inter-group divisions [54, 55].

This form of polarization is linked to a different understanding of politics, in which the causal arrow of politics is reversed; in this model, politics moves from partisan belonging, to policy opinions, to arguments for these opinions. As Achen and Bartels [27] put it, “voting behavior is primarily a product of inherited partisan loyalties, social identities and symbolic attachments. Over time, engaged citizens may construct policy preferences and ideologies that rationalize their choices, but those issues are seldom fundamental.” Such *post hoc* rationalization of views has been an important focus in recent years of social psychology and political science research, emphasizing how our identity shapes our psychology and cognition through various mechanisms. For instance, “confirmation bias”, “deductive” and “motivated reasoning”–in which our objective judgment and our rationality is affected by our identities and interests—and the “backfire effect” where an argument against our position causes us not to loosen our view, but to dig in, and strengthen it [56–60].

Such an “Identity-Protective Cognition” [61] has been described as a way to avoid dissonance and estrangement from our social groups by subconsciously resisting information that could threaten our defining values. Arguments against our “cherished beliefs” [62] have been shown to activate the same neural paths as the threat of physical violence [63], and the secretion of higher levels of cortisol in saliva, indicating stress [64]. When identities are the dominating force of politics, reasoning is thereby less of a foundation for political belonging, and more of an expression of it [61, 65]–as Schmitt [66] succinctly puts it, “it’s not what you say about the issues; it’s what the issues say about you.”

A shift toward a strengthening of partisan identity has meant that partisan identity is coming to absorb a broader spectrum of social identities, thereby aligning “otherwise unrelated divisions, emasculating cross-cutting cleavages, and dividing society and politics into two separate, opposing, and unyielding blocks” [1]. This has brought the diverse social identities of plural societies to coalesce into a singular split between two “mega-identities” [67], with partisanship growing in importance also outside the political arena. Partisan affiliation not only shapes political behavior [68], but is also expressed for example in where we live [69–71], what car we drive [72], who we choose to make our partner [73, 74], and much more [17, 46, 75, 76]. As research using survey data has shown, this means that opinions are not so much diverging as they are becoming clustered and interlinked [77].

Polarization thereby becomes “a process whereby the normal multiplicity of differences in the society increasingly align along a single dimension, cross-cutting differences become reinforcing, and people increasingly perceive and describe politics and society in terms of ‘us’ versus ‘them’ [78]. The polarization that affects many democracies today is thus seen as resulting from strengthening political identities that come to dominate pluralistic social life, to channel and absorb other cleavages, while effacing countervailing identities [1]. The outcome is a sense of fundamental difference and a mutual questioning, or even denial, of the other’s legitimacy—more akin to an ethnic separation than to a difference in opinion [18].

To summarize, the identity-driven explanation of political polarization suggests that the current wave of polarization around the world is the result of a shift to a state where partisan identity has become more important as driver of political life. In this view of politics, polarization is not a result of diverging opinions and a rational exchange of arguments, but rather of the ascendance of a different type of dynamics that transforms the underlying social psychological substrate of politics. This calls for a corresponding new hypothesis of social media polarization that can explain the observed strengthening partisan identities, from the standpoint of the social-psychological micro-dynamics of social identity. Ahead of formulating such a model, this is the field towards which we now turn.

### The social psychology of intergroup conflict

Decades of social-psychological research has shown that group identity balances between two counteracting forces, represented by two frameworks of social psychology. The minimal group paradigm represents separating social forces, while contact theory represents integrating social forces [8, 52, 53].

The central finding of the minimal group paradigm [52, 53, 79] is that when individuals come together to interact under the label of a similarity, this similarity will tend to turn into a social identity. Even the most arbitrary token can be coopted as the symbolical foundation of a strong social identity. In one famous experiment, groups were formed on the basis of how participants answered the question “who is your favorite abstract expressionist?” As peripheral as the question itself was to participants, it was still effective as an identity marker that separated an “us” from a “them” and that thereby catalyzed a dynamical deterioration into conflict and polarization [80]. Another important finding of this literature is that is that if individuals have a choice they will tend to self-segregate into groups on the basis of who they are most similar to. As Gordon Allport [81] argues, people automatically tend to spend time with people like themselves, as “it requires less effort to deal with people who have similar presuppositions”. This suggests an ever-present latent drive toward the formation and polarization of social groups.

Contact Theory points to the counteracting forces, which tend to integrate groups and potentially reduce intergroup conflict. Contact theory goes back to the work of Gordon Allport [81], who argued that interaction between groups, given the right conditions, can reduce group separation and prejudice. When groups meet in (1) the presence of intergroup friendships, (2) the absence of anxiety, and (3) the presence of empathy, group boundaries can start to dissolve. Even indirect contact, such as mediated exposure to another group, or even mediatized representation of ingroup friendship with an outgroup member, can under certain circumstances reduce prejudice between groups, as has been shown by significant research under the label of the “parasocial contact hypothesis” [82–84]. When opposing groups meet without these conditions being fulfilled, however, the meeting can instead lead to expressions of conflicts, which further intensifies the polarization.

Between them, these two theories describe social identity as balancing between centrifugal and centripetal forces. While there is an inherent social tendency toward fragmentation and tribalization, this tendency is counteracted by the integrative action of many of the institutions that surround us. The army was the example *par excellence* of such a meeting-place, with significant research showing its integrative capacities [53, 81]. The workplaces, neighborhoods, everyday institutions—they all fulfill the conditions of contact theory to the extent that they cause us to come together to achieve a common goal.

This paints the outlines of an alternative mechanism to the echo chamber to account for the link between new digital media and polarization: increased opportunities for self-segregation does not primarily lead to diverging and extreme opinions by limiting exposure to competing viewpoints, but by shifting the balance between the centrifugal and centripetal forces of social identity. New media simply relaxes the external constraints that have historically introduced the integrating forces understood via contact theory, such as neighborhoods, workplaces, villages, etc. Rather than meeting under the label of a geographical location, new information technology enables us to increasingly meet under the label of shared interests or attributes—and meeting under the banner of shared interests and attributes leads to the formation and strengthening of social identities [85, 86].

## Model description

The model formally implements the balancing forces of identity- and group formation described above as *the minimal group paradigm* and *contact theory* (see *The social psychology of intergroup conflict*). The aim is to enable an exploration of how social identity behaves on the societal level as we change the flexibility of social interaction to mimic the effects of new information technology.

Following this social identity paradigm, the model suggests that as we interact with others, we receive social feedback that leads us to either strengthen or weaken our expressed identity. Simply put, the model suggests that new media technology shifts our interaction from being primarily local—we interact with those who are spatially near us—to being less constrained by space, which gives us more freedom to come together with others who are similar to us. As we do so, our shared attributes come to strengthen into salient social identities. This reduces our shared ground with those who differ from us, resulting in that the interactions that do take place become more conflictual.

Agents receive and provide feedback in the form of social responses to expressed identities. Based on this feedback they adapt their behavior using a reinforcement learning approach [87]. Each agent holds one out of a number of possible identities. They prefer to interact with agents that identify similarly to themselves, but are constrained by exogenous factors (e.g., workplaces, neighborhoods, physical distance, and search costs) from choosing entirely freely. We model the effects of changes in communication technology by varying the strength of this constraint.

**Fig 1:**
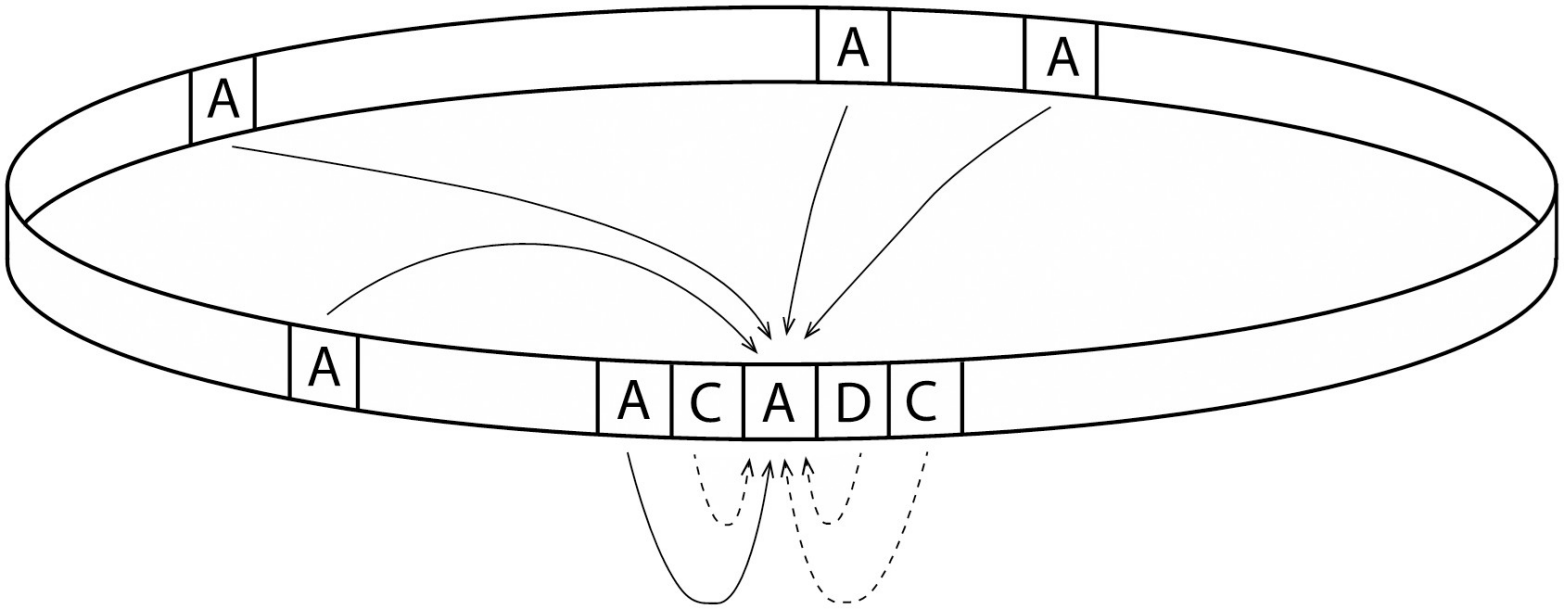
Model illustration. An illustration of the model, showing four *local* and four *global* connections of an individual with identity A. Positive feedback is received from connections to individuals with the same identity (solid) and negative feedback from those to dissimilar identities (dashed). Local connections represent externally constrained interactions (geography, workplace, etc.) and may be heterogeneously positive and negative. Global connections represent interactions afforded by technology. These are selected to be positive and are thereby homogenously positive. The magnitude of the feedback is the product of the strength of the identity of both individuals.

The model is formulated as follows. We have *N* agents and *M* possible identities. Agents *a*_*i*_ (with *i* = 1, …, *N*) have an identity *k*_*i*_ ∈ (1, ‥, *M*) held with strength ${\tilde{w}}_{i}\in [0,\infty ]$. For clarity of representation, the identity strength is scaled to the interval [0.1] using $w_{i}=1\;–Exp(-{\tilde{w}}_{i}),\;$ whose value is furthermore constrained from the extremes so that *w*_*i*_ ∈ [*w*_*min*_, *w*_*max*_], where *w*_*min*_ = 0.05 and *w*_*max*_ = 0.95. (Using *w*_*min*_ = 0 and *w*_*max*_ = 1 may too easily result in fixation in the learning process: the former corresponds to having an identity without any strength whereas the latter corresponds to infinite strength.) As illustrated in Fig 1, each agent has a position in a one-dimensional lattice with periodic boundary conditions. The model also includes a slow random migration to introduce exogenous variability in social contexts—agents randomly swap places with probability *ϵ* = 0.02. With this mechanism we avoid the establishment of areas of strong identities that stem from the initial setting of identities (that may happen to be dominant in one identity). Since the migration is random, we also avoid the mechanism that characterizes the segregation in Schelling-type models [88].

Agents are picked at random to interact with *n*_*loc*_ of the closest neighbors on the lattice (exogenously constrained interactions), and *n*_*glob*_ globally selected agents with whom it shares identity (interactions by preference). The ratio *n*_*loc*_/*n*_*glob*_ thereby models change in the constraint on freely choosing interaction partners, and its effect on the relative importance of similarity (see Fig 1).

The utility that an agent *a*_*i*_ derives is summed over the *n*_*loc*_ + *n*_*glob*_ agents *a*_*j*_ with which it interacts. If *a*_*j*_ holds the same identity as *a*_*i*_ (i.e., *k*_*i*_ = *k*_*j*_) the agent gets a utility contribution of *w*_*i*_*w*_*j*_, that is, the combined (scaled) identity strengths of *a*_*i*_ and *a*_*j*_. That is, finding someone with a shared identity, for whom this identity is important, is rewarded with a strong positive feedback. Similarly, when an identity is presented and the responding agent does not share this identity, a negative response of −*w*_*i*_*w*_*j*_ will be given. This results in an aggregate utility *u*_*i*_ as the sum of all contributions from the interactions with other agents,

$$u_{i}=\sum_{j\in S_{i}}\;{w_{i}w}_{j}(2\delta_{k_{i}k_{j}}-1).$$ (1)

Here $\delta_{k_{i}k_{j}}=1$ if identities are equal (*k*_*i*_ = *k*_*j*_), otherwise $\delta_{k_{i}k_{j}}=0$. *S*_*i*_ is the index set of the agents with which *a*_*i*_ interacts.

This utility *u*_*i*_ is then used to update the agent’s identity strength, $w_{i}^{\prime }=w_{i}+\;\Delta w_{i}$, using an adaptation step, similar to reinforcement learning. A positive utility results in an increasing identity expression, and vice versa. Expressed in the non-transformed strength, ${\tilde{w}}_{i}$, the identity change is proportional to the utility,

$$\Delta {\tilde{w}}_{i}=d\;u_{i},$$ (2)

which is modified to the following expression in the transformed strength *w*_*i*_,

$$\Delta w_{i}=d\;u_{i}(1-w_{i}),$$ (3)

with a learning rate constant *d* = 0.5. Note that the factor (1 − *w*_*i*_) comes from the scaling of the strength to the unit interval. (Differentiation of $w_{i}=1\;–Exp(-{\tilde{w}}_{i})$, results in ${dw}_{i}=(1\;–w_{i})d{\tilde{w}}_{i}$, which in turn gives Eq (3). This mean that the result of learning on change of an identity strength closer to 1 is damped due to the scaling factor.) Identity strengths are initialized to the lowest strength value *w* = 0.05.

In addition to the learning mechanism, noise is introduced so that agents perform small explorative changes of identity strength. With a certain probability, *p*_*expl*_, a small explorative change of δ*w*_*i*_ = (1 − *w*_*i*_)*w*_*expl*_ is added to the strength in the learning step; this is (approximately) equal to an explorative strength change *w*_*expl*_ in the non-transformed strength $d{\tilde{w}}_{i}$. We have used the probability *p*_*expl*_ = 0.2 and *w*_*expl*_ being a uniformly distributed random value in the interval [−*c*_*expl*_, *c*_*expl*_]. We have chosen *c*_*expl*_ = 0.05 i.e., an exploration of a strength change at the same level as the minimum strength value.

## Results

**Fig 2:**
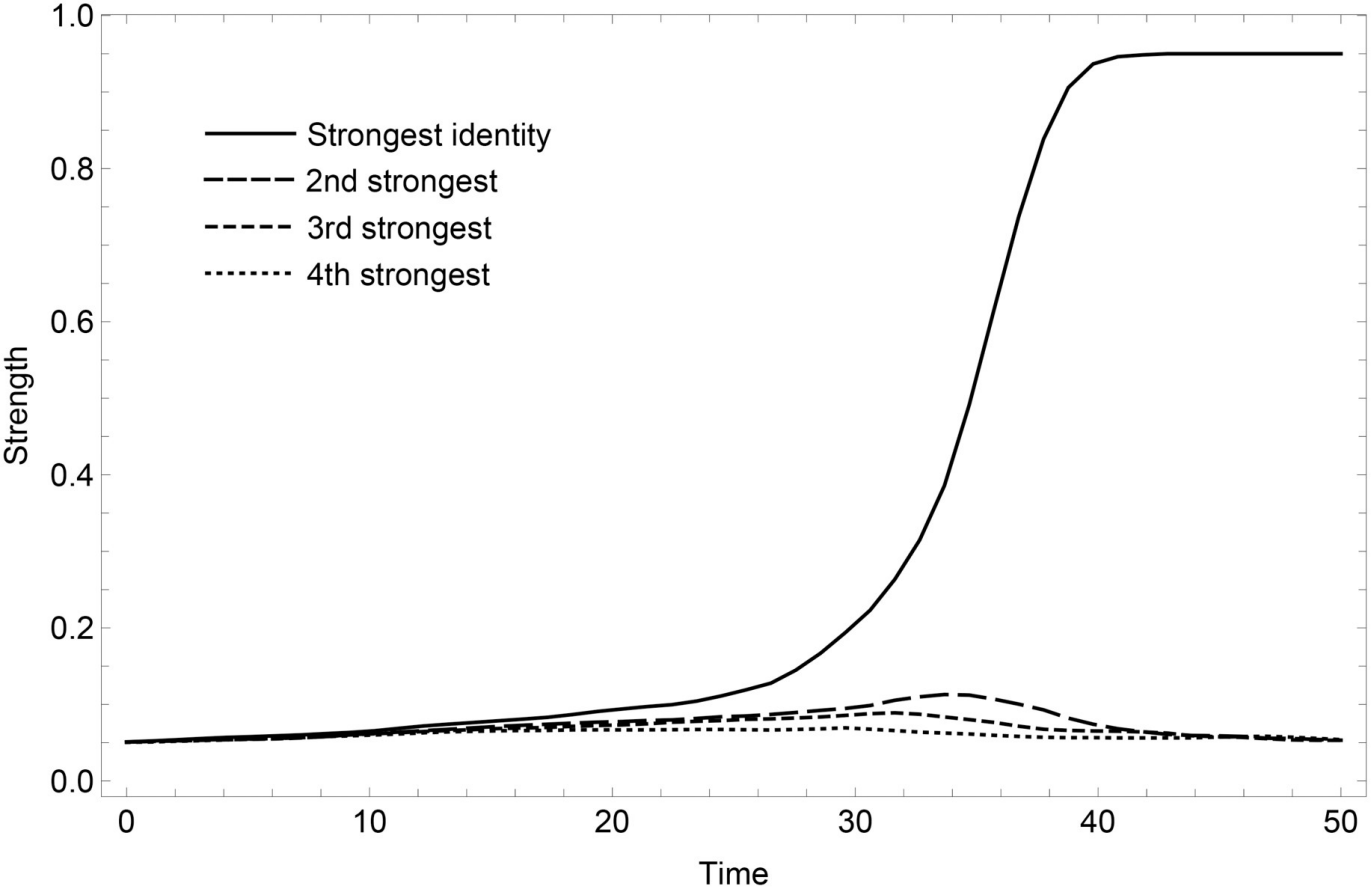
Transition. The transition to a dominating identity is illustrated in a simulation where individuals have 3 global and 5 local connections. In each time step, the average strengths of the 4 possible identities are plotted. The simulation is based on a system of N = 200 individuals, and 50,000 individual learning updates are applied. The M = 4 different identities are represented by full to dotted lines in their order of dominance.

Fig 2 illustrates the basic behavior of the model. Random initial fluctuations, including identity exploration, in local neighborhoods are dynamically self-reinforced by the learning dynamics. This causes one or more identities to become fixated near the maximum expression, while the other identities are suppressed and remain near the minimum level of expression.

**Fig 3:**
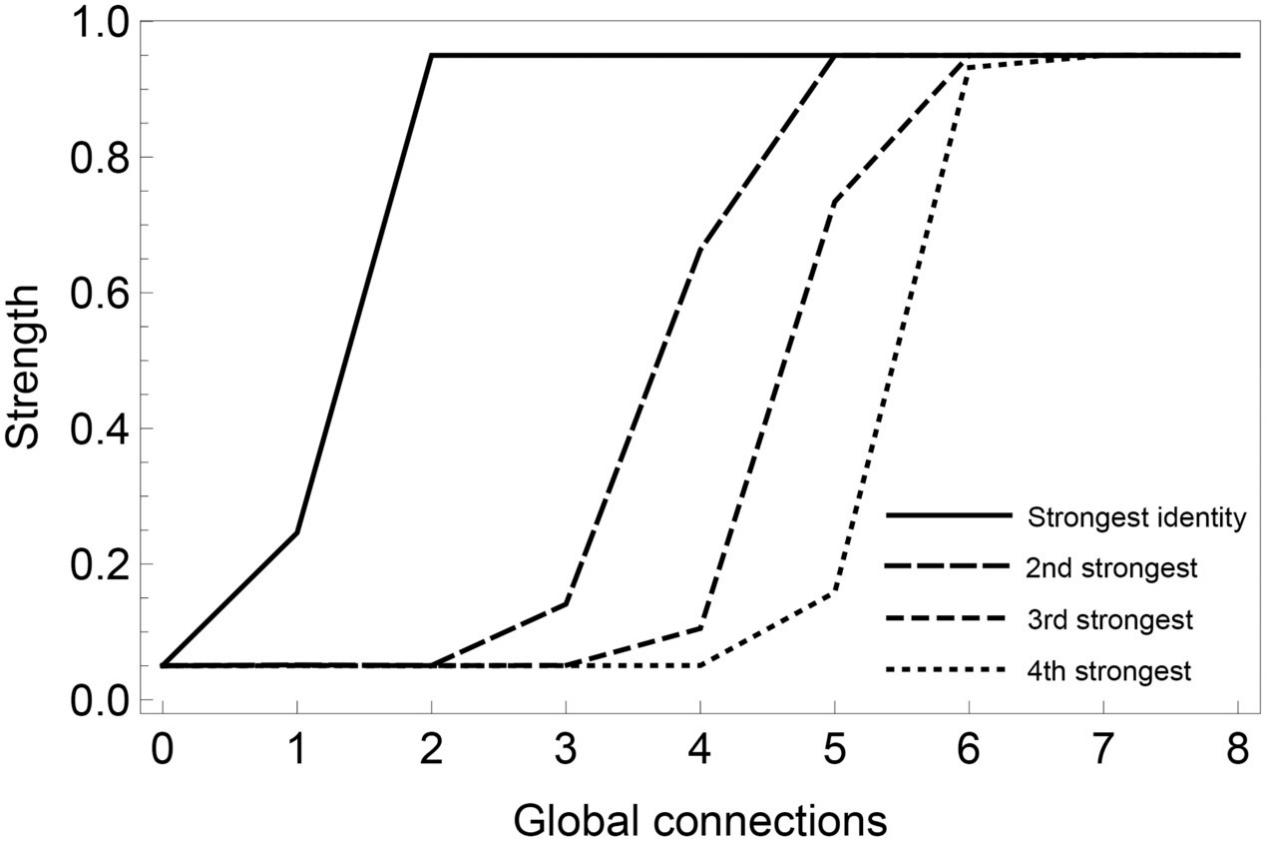
Transitions between regimes. Transitions to one or several dominating identities are shown as a function of the number of global connections (*n*_*glob*_). The plot is based on 50 simulations for each value of *n*_*glob*_, with averages of final strength values plotted in the graph. We use N = 200 individuals and each run is 200,000 time steps, i.e., individual reinforcement learning steps. For each run, the M = 4 different identities are represented by full to dotted lines in their order of dominance. The graph illustrates that, when the local neighborhoods are dominating (smaller number of global connections), only zero or one of the identities develops towards high identity strengths. When connections can be chosen freely (larger number of global connections), a larger number of the identities achieve higher strength values.

Fig 3 examines the outcome of increasing the number of global connections *n*_*glob*_ (decreasing the *n*_*loc*_/*n*_*glob*_ ratio) to simulate an increasing technology-induced freedom to choose with whom to interact. The total number of connections is kept constant, *n*_*loc*_ + *n*_*glob*_ = 8 [e.g. 89]. If all connections are local, i.e., *n*_*glob*_ = 0, the model settles to an overall low level of identity strengths. Single identities may at times become stronger, since they may be shared by local groups, but this effect is weak and kept at bay by migration. In other words, the centripetal forces represented by contact theory here dominate. When *n*_*glob*_ increases, these fluctuations in identity strength will be more and more likely to undergo the dynamical self-reinforcement that we observed in Fig 2. With an increasing number of global connections, an increasing number of dominating identities can co-exist at steady-state. As expected, for sufficiently many global connections, all identities will increase their strengths to the maximum level. What we see is an ascendance to dominance of the centripetal force represented by the minimal group paradigm.

**Fig 4:**
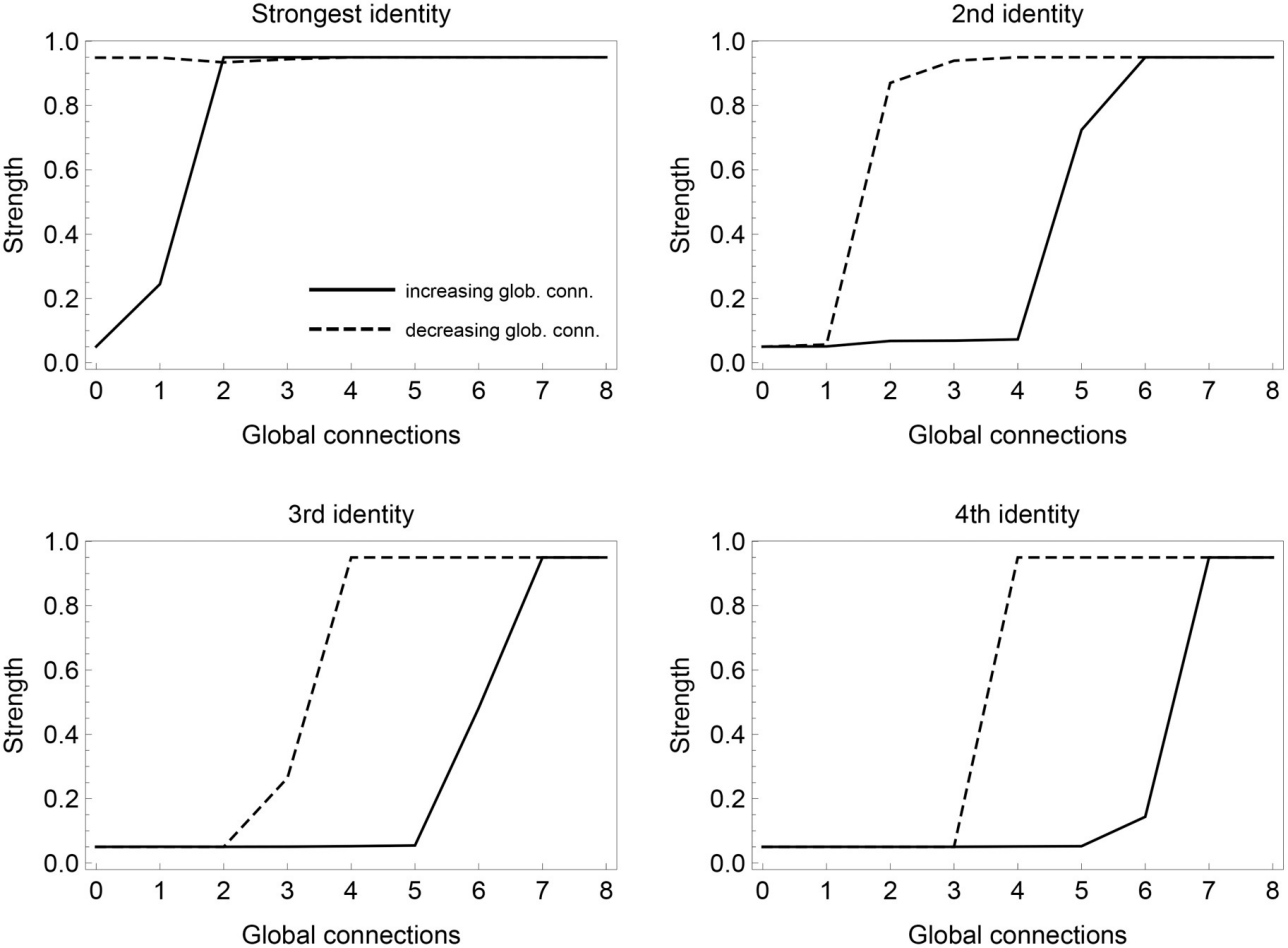
Hysteresis. Hysteresis is illustrated by the difference in transition points, between weak and strong identities, when increasing (full line) and decreasing (dashed line), the number of global connections. The panels are based on a model experiment in which we (i) start with 0 global connections, (ii) let the identity dynamics run to an essentially stationary situation and store the strengths of the 4 different identities, and (iii) from this situation the global connections is increased by 1, and the identity dynamics continues, and so on. This is continued up to 8 global connections, after which the procedure is continued, but with a decreasing number of global connections down to 0. The identity strengths are calculated as averages from 50 simulations. The different panels **(a-d)** show the identity strength for the strongest identity **(a)** to the weakest identity **(d)**. The full lines show the average strengths when we have an increasing number of global connections, while the dashed lines show the average strengths when going back with decreasing number of connections. As in Fig 3, we see that the more global connections, the more identities may develop to a maximum expression level. It is clearly seen that transitions back to lower expression levels do not occur until significantly lower values of *n*_*glob*_ are reached, and for the strongest identity **(a)** it does not even happen at *n*_*glob*_ = 0. The size of the system is N = 200 individuals.

Fig 4 examines historical path-dependence in the model by varying *n*_*glob*_ within the same historical trajectory. This is intended to mimic historical innovation of increasingly effective information technology.

This model experiment is set-up as a number of runs in which each run follows a sequence of stepwise increases of global connections followed by a corresponding decrease sequence. Each run is described as follows: (i) start with 0 global connections, (ii) let the identity dynamics run to an essentially stationary situation (200,000 individual learning updates) and store the strengths of the M = 4 different identities (sorted by their strengths), and (iii) from this situation the global connections (*n*_*glob*_) is increased by 1, and the identity dynamics continues (with another 200,000 updates), and so on. This is continued up to 8 global connections, after which the procedure is reversed, with a decreasing number of global connections down to 0. The identity strengths for each value of *n*_*glob*_ are then calculated as averages from 50 simulations of the full process described here.

The four panels (a-d) of Fig 4 represent the 4 different identities, sorted in the order of their strengths from strongest to weakest. When we increase the number global connections, following the full lines in Fig 4, an increasing number of identities become dominant, as we saw in Fig 3, but here it happens for lower values of *n*_*glob*_ since we do not start anew from the initial mixed state with each increment. If we reverse history to reduce the fraction of global connections from a high level, following the broken lines in Fig 4, we see that the system does not readily go back to a situation with fewer dominating identities. We see that even when we get down to a fully locally dominating neighborhood (*n*_*glob*_ = 0), there is always a remaining dominating identity as a remnant from a situation in which several global connections were present. The system, in other words, exhibits hysteresis, i.e., the effects are not symmetric. This implies that even if the effects of new media were reversed, and social interaction became more locally constrained, the system would not return to its weak-identity state. The system is in this sense locked into its polarized state, which may be highly challenging to reverse.

The robustness of this result was tested in a sensitivity analysis in which several parameters were varied. The most critical one is the rate of random migration, which counteracts the formation of local clusters of strong identities. When the migration rate is reduced from 0.02 to 0.005, one can observe that the hysteresis phenomenon is starting to break up. For details on the sensitivity analysis, see S1 Appendix.

## Discussion

In this paper we have introduced a simple formal model based on social psychological mechanism that explores affective polarization on the societal level. We have observed a host of emergent phenomena that are not accessible for theoretical (and ultimately empirical) exploration without the use of simulation [90].

The major finding is that the interplay between social identity and the structure of social interaction produces two distinct and dynamically stable regimes on the macroscopic level. One *fluid* regime in which identities are weak and social connections heterogeneous, and a second *solid* regime in which identities are strong and groups homogeneous. We interpret the latter as a state dominated by partisan identity will tend to be self-maintaining via feedback mechanisms. Political issues will continue to be sucked into the realm of group identity in a process that transforms the substance of democracy—i.e., facts, arguments, opinions, and so on—into the substance of tribalism.

Moreover, we find that the fluid and solid regimes are separated by a sharp transition. We propose that the mechanism of this transition is the same that has been observed in political polarization by scholars in recent decades [10]. In other words that an increased freedom to interact with similar individuals leads to strengthened and aligned social identities, which then further increases the preference for homogenous interactions. The model tells us that this process appears to create a tipping-point effect on the societal level. Beyond the tipping point, the integrating forces represented by contact theory are overwhelmed and polarization enters a self-reinforcing spiral.

Finally, we observe hysteresis in the dynamics. This means that returning to previous levels of heterogeneous interactions in the solid regime will not easily return us to the fluid regime (see Fig 4). Reducing polarization is harder than simply learning to get along within frameworks that worked in the prior regime. The process itself changes these frameworks, and so returning to an equivalent state (in terms of restoring the function of rational debate) would require new political frameworks that adapt our political models and processes to this new situation.

## Conclusion

From a methodological horizon, this paper has taken some important initial steps toward using simulation as a way of exploring and testing the adequacy of hypotheses about the link between micro-level social psychological processes and the societal phenomena that they cause [90]. These phenomena are riddled with feedback effects and nonlinearities, and thereby stand to gain from a formal modeling approach to capture the complex dynamics of social identity within political life. The model presented here resembles formal models of opinion dynamics, and thereby brings into dialogue the literature on opinion dynamics with the emerging research on affective and social polarization.

The macroscopic patterns that we have observed in the model dynamics are consistent with the type of political transition that has been observed by affective polarization scholars over recent decades. That is, a transition where partisan identity becomes the driver of politics, and where other social identities coalesce under partisan identity. Our study contributes to this picture a set of dynamic phenomena that arise on a societal and historical scale, namely the formation of distinct regimes of system behavior, thresholds, and hysteresis.

More specifically, we suggest a novel possible causal link between the development of new information technology and the contemporary rise of affective political polarization. The suggestion is that the causal link is not about the political views of individuals becoming more extreme due to lack of exposure to alternative viewpoints, but that new media technology is part of a larger scale sociotechnical shift in society, which has shifted the balance between the centrifugal and centripetal forces of social identity, causing a strengthening of the role of identity in political life. This shifts the focus from the micro- and meso-levels of individual and group radicalization via online echo chambers, to the larger-scale societal shift of which social media is part.

We see information technology as part of a larger sociotechnical shift that has been observed by scholars from diverse traditions; processes of specialization, fragmentation, and segregation, of which new media technology is as much an expression as a cause [91–93]. New media technologies are part of an information and innovation economy, emphasizing economy-of-scope and smaller, more homogeneous work groups compared to the large-scale industrial production of old. This shift is taking place in both offline and online environments and can therefore not be meaningfully assessed through a comparison between the two. Its effects and causes will play out in all facets of social life, and be reflected in the type of strengthening and clustering of identities that studies have indeed identified [71, 94]. Importantly, this implies that we could look at political polarization at yet a higher level of analysis, namely as a shift in the structure of identity in society.

Our model suggests that affective polarization is part of a dynamics with two stable regimes that we have referred to as fluid and solid. The fluid regime is characterized by heterogeneity of interaction and weak social identities. The solid regime is characterized by the homogeneity of interaction and strong social identities. The fluid state recalls Durkheim’s [95] notion of “organic solidarity”, where society is held together by differences and mutual dependence, while the solid regime recalls his concept of “mechanical solidarity”, where groups finds their cohesion and integration in homogeneity.

Politics in the fluid regime then emerges out of a situation where identities within the population are weak, and interactions are primarily shaped by external factors, such as geography, workplaces, and so on. Social identities are not strong enough here to coopt political opinions. Neither are they strong enough to undermine mutual trust in the population, so rational debate remains viable as a mechanism for resolving differences of opinion, and remaining differences will not have strong social consequences. These are not societies in which conflict is absent, but rather in which the conflicts of a plural society are cross-cutting, meaning that particular social groups were allies in some circumstances and opponents in others. As scholars have long argued, social conflict is sustainable as long as there are multiple and nonoverlapping lines of disagreement, meaning that “segmental participation in a multiplicity of conflicts constitutes a balancing mechanism within the structure” [96].

Politics in the solid regime represents a condition where partisan identity strengthens and begins to align with other social identities. The model has shown a scenario in which new information technology triggered such a transition by facilitating the social psychological processes that form and strengthen social identity. The model found that due to systemic feedback effects, once the system passes a certain tipping-point of polarization, the system can go into run-away political polarization driving a transition to the polarized strong-identity regime, which may be difficult to reverse due to hysteresis.

More broadly, examining the effects of digital media on polarization through the lens of the social identity literature suggests that these effects are more pernicious than suggested by the common understanding of the echo chamber hypothesis. Polarization transforms the very substance of rational debate—such as opinions, arguments, and values—into symbols of identity. This means that debates lose part of their capacity to resolve conflicts, as they become founded not in rational deliberation, but on the dynamics of social status and intergroup conflict [61, 97]. The suggestion here is not that no rational debate is taking place, but that the increasing dominance of political identity undermines the possibility for their resolution. In other words, contrary to the echo chamber hypothesis, which sees polarization as taking place within the space of opinions, we paint a picture where opinions become less and less salient *as* opinions, while they become more and more salient as markers of identity.

This insight can also help resolve important dilemmas facing the traditional echo chamber hypothesis. The mainstream echo chamber hypothesis tends to be understood through the dictum *homogeneity breeds extremism*. This hypothesis has however been increasingly questioned as empirical studies find that some digital spaces are in fact characterized by significant interaction across the political divide, which—contrary to expectations—does not appear to be reducing polarization [42, 98–101]. Going to the literature on social identity provides a potential solution, by modifying the echo chamber dictum to *homogeneity strengthens social identity*. As the literature on social identity suggests, this means that forcing interaction between opposing groups is likely to have limited effects on reducing polarization—some studies even suggest that doing so can result in a backfire effect, in which interaction between opposing groups strengthens polarization even further [102]. Social media may thus be polarizing not by enabling us to completely avoid opposing views, but by providing spaces for both isolation and conflictual interaction. Some digital spaces, such as dedicated discussion forums, subreddits or private Facebook groups, enable us to meet under the banner of a shared attribute, which can thus develop into a social identity, as described by the model in this paper. While other digital spaces, such as Twitter or Facebook, may drive interaction between opposing groups, this is unlikely to help de-escalate identity polarization, but may even exacerbate it—becoming akin to throwing individuals into the midst of a political war, forcing them to pick sides, and thus transforming the social identities into the substance of intergroup conflict. The model fits into a broader paradigm on social identity which suggests that polarization is the result of a *combination* between group isolation and interaction: the former develops an ingroup social identity, that the latter channels into intergroup conflict [101, 103]. Future studies may be directed to further explore the implications of this line of research on social media polarization.

Future research may furthermore seek to integrate this model with previous work on the clustering of identity, such as DellaPosta et al [17], to show that this shift also produces the observed sorting of identities [11]. Another direction is to investigate the interplay between social identity and rationalistic opinion dynamics. How would the ability to identify and solve problems deteriorate under the idea that identity not only governs the structure of the social network substrate of the latter, but that it also, so to speak, preys on the latter by coopting its substance as symbols of identity.

The model at hand furthermore leads to some testable predictions. First, political polarization should be linked to increasing scale of political culture—from local to national—as the model suggests a link between the intensity and scale of political conflict. Secondly, the paper suggests that the interaction between ideological groups that has been observed in the empirical literature on echo chambers [42, 98–100] is characterized not by rational exchange of arguments, but rather by conflictual interaction serving to signal group belonging.

## Supporting information

S1 Appendix (DOCX)
