# Supplementary material for: Modeling the emergence of affective polarization in the social media society
Source: PLoS One. 2021 Oct 11;16(10):e0258259. doi: 10.1371/journal.pone.0258259 (PMC8504759; doi:10.1371/journal.pone.0258259)
Supplement: S1 Appendix — (DOCX) [file pone.0258259.s001.docx]

# Supporting Information S1 Appendix

In order to investigate the model robustness to changes in parameter values, we have performed a sensitivity analysis in which the hysteresis diagrams are plotted for various modifications of parameters.

**(b)**

**(a)**

$$m = 0.01$$


**(d)**

**(c)**

$m = 0.$005

$$m = 0.002$$

**S1 Fig. 1. Sensitivity analysis - migration rate.** The migration rate $m$ is a critical parameter since it controls the mechanism that breaks up local clusters of strong identities. The hysteresis phenomenon is investigated for reduction of the migration rate, from the setting of $m = 0.02$ in the main text, shown in panel (a) for comparison, to lower levels: $m=0.01$ in panel (b), $m=0.005$ in panel (c), and $m=0.002$ in panel (d). For the strongest identity, the hysteresis effect breaks up at $m=0.005$. (Experiment set-up as in main text, but only 10 runs per data point.) As is seen in this figure, the hysteresis phenomenon is starting to break up when $m$ is reduced from $0.02$, the setting used for the investigation in the main text, to $0.005$, at least for the strongest identity; cf. panels (a) and (c).

**(b)**

**(a)**

**(c)**

**(d)**

**S1. Fig 2. Sensitivity analysis - other parameters.** The hysteresis phenomenon is investigated for changes in parameter settings. Panel (a) for the main text setting, c.f. regarding (b) exploratory strength changes $c_{expl}$, (c) the strength limits, and (d) the total number of connections, all in comparison with the main text result shown again in panel (a). (b): Reducing $c_{expl}$ from $0.0$5 to $0.01$ does not change the result. (c): Changing the strength limits from [0.05, 0.95] to [0.01, 0.99] results in a stronger hysteresis effect, primarily since it will be more difficult for low strength identities to increase their strength. (d): Increasing the number of connections from 8 to 12 leads to a similar hysteresis pattern. (Experiment set-up as in main text, but only 10 runs per data point. For panels (c) and (d) each run involves 400,000 learning updates.) Changing the exploration constant $c_{expl}$, that sets the interval size for random explorations of strength changes does not seem to have a strong effect on the hysteresis phenomenon. In the main text investigation, we have limited the (transformed) strength values to $[0.05, 0.95]$. (For this test 400,000 learning updates have been used.)
